# Staged replacement of both hips and both knees in patients with rheumatoid arthritis

**DOI:** 10.1186/s12891-023-06282-4

**Published:** 2023-03-28

**Authors:** Jian Cao, Wenqi Wang, Wei Feng, Hong Xu, Duan Wang, Zongke Zhou

**Affiliations:** 1 Department of Orthopedics, Orthopedic Research Institute, West China Hospital, Sichuan University, No.37, Guoxue Road, Wuhou District, Chengdu, 610041 Sichuan People’s Republic of China; 2 West China School of Medicine, Sichuan University, No.37, Guoxue Road, Wuhou District, Chengdu, 610041 Sichuan People’s Republic of China

**Keywords:** Bilateral, Four joints, Cementless total hip replacement, Cemented total knee replacement, Rheumatoid arthritis, Outcomes

## Abstract

**Background:**

Patients with rheumatoid arthritis (RA) undergoing bilateral total hip arthroplasty (THA) and total knee arthroplasty (TKA) are an uncommon population, and their outcomes are also difficult to predict. The purpose of this study was to evaluate whether both bilateral cementless THA and cemented posterior-stabilized TKA (PS-TKA) can provide reliable outcomes for RA patients.

**Methods:**

We retrospectively reviewed 30 RA patients (60 hips and 60 knees) who underwent both elective bilateral cementless THA and cemented PS-TKA, with a minimum follow-up of 2 years. Clinical, patient-reported, and radiographic data were retrospectively analyzed.

**Results:**

The mean follow-up was 84 months (range, 24–156). By the last follow-up, the post-operative range of motion, Harris Hip Score, Knee Society Score (KSS) clinical, KSS functional, Western Ontario and McMaster Universities Index of Osteoarthritis (WOMAC) hip, and WOMAC knee scores were significantly improved compared to the preoperative values. All patients achieved the ability to walk. In addition, overall satisfaction scores on a 100-point scale were 92.5 after THA and 89.6 after TKA. Only one patient underwent revision surgery due to knee joint instability, and all replaced hips and knees were radiographically stable by the assessment of the radiolucent line. The proportion of implants that did not suffer loosening or require revision surgery was 99.2% during the 84-month follow-up, based on Kaplan-Meier analysis.

**Conclusions:**

Our study suggests that bilateral cementless THA and cemented PS-TKA provides reliable mid-long-term clinical, patient-reported, and radiographic outcomes in RA patients, with high survivorship and patient satisfaction.

## Background

The design of prostheses and the corresponding surgical techniques have significantly advanced in recent decades [1, 2], which makes total hip arthroplasty (THA) and total knee arthroplasty (TKA) become two routine surgical procedures to relieve pain, correct deformities, and restore physical function for patients with end-stage hip and knee diseases. However, performing bilateral THA and TKA (BTHKA) on the same patient is uncommon, complex, and time-consuming, and the outcome is difficult to predict [3–5]. Indeed, the patient may demonstrate deterioration in some aspects of function undergoing the operation, especially in patients with rheumatoid arthritis (RA), who show varying degrees of pain, deformity, and dysfunction in the upper limbs, and who are meanwhile at significant risk of revision surgery [3, 6, 7].

To our knowledge, only seven studies have explored the outcomes of BTHKA in RA patients (Table 1); these studies are limited by relatively small samples [3, 8–11], short mean follow-up [3, 7–10], and limited radiographic follow-up [3, 6, 7, 9–11]. All studies were published at least 30 years ago, and they used diverse outdated prosthetic designs and surgical techniques. In addition, although patient-reported outcomes measures (PROMs) have become a criterion standard as a measure of outcome in orthopedic operation [12], most did not quantify patient-reported outcomes of BTHKA using validated hip- and knee-specific scores [6, 7, 9, 10].

**Table 1:** Reported outcomes of bilateral total hip and knee arthroplasty in patients with rheumatoid arthritis

Study	Year	Patients	Mean age (range)	Hip prostheses	Knee prostheses	Radiolucent line	None or mild pain		Flexion (°)			Unable walking	Walking aids	AL	PJI	Survival	Follow-up (months)
							Hip	Knee	Hip	Knee	Combined						
Johnson et al. [8]	1975	11	51 (21–70)	Two types Outdated	Two types Outdated	NA	11/11 (100.0%)	11/11 (100%)	80	78	158	0/11 (0%)	6/11 (54.5%)	0	0	100%	27 (19–58)
Jergesen et al. [3]	1978	16	55 (22–72)	There types Outdated	Six types Outdated	NA	15/16 (93.8%)	15/16 (93.8%)	105	100	205	0/16 (0%)	10/16 (62.5%)	0	0	96.9%	22.8 (9–42)
McDonald et al. [7]	1982	26	46 (24–62)	Two types Outdated	Nine types Outdated	NA	20/26 (76.9%)	13/26 (50%)	91	90	181	0/26 (0%)	17/26 (65.4)	4	1	96.2%	25 (7–63)
Yoshino et al. [9]	1984	18	56 (37–69)	Three types Outdated	Four types Outdated	NA	18/18 (100%)	13/18 (72.0%)	82	82	164	2/18 (11.1%)	18/18 (100%)	1	0	98.6%	33 (12–120)
McElwain et al. [10]	1985	19	54 (27–67)	One type Charnley prostheses	One type Sheehan prostheses	NA	19/19 (100%)	17/19 (89.5%)	98	99	197	2/19 (10.5%)	13/19 (68.4%)	0	0	100%	27 (6–84)
Suman et al. [6]	1986	20	59 (49–78)	Four types Outdated	Five types Outdated	NA	19/20 (95%)	15/20 (75%)	100	97	197	0/20 (0%)	14/20 (70%)	3	1	95.0%	Not available (24–156)
Hoekstra et al. [11]	1989	13	45 (18–68)	Six types Outdated	Three types Outdated	NA	13/13 (100%)	13/13 (100%)	79	81	160	1/13 (7.7%)	6/13 (46.2%)	1	0	98.1%	71 (32–92)
Current study	2022	30	47.2 (26–79)	One type Cementless prostheses (DePuy)	One type Cemented posterior-stabilized fixed-bearing prostheses (DePuy or Stryker)	Radiographically stable	30/30 (100%)	30/30 (100%)	114.5	104.1	218.6	0/30 (0%)	11/30 (36.7%)	0	0	99.2%	84 (24–156)

*AL* Aseptic loosening, *PJI* Periprosthetic joint infection, *NA* No assessment

Therefore, we retrospectively evaluated whether BTHKA with relatively uniform hip and knee prostheses can provide reliable clinical, patient-reported, and radiographic outcomes for RA patients.

## Methods

This study was approved by our institutional Ethics Committee. A total of 33 consecutive RA patients who underwent elective bilateral cementless THA and cemented posterior-stabilized TKA (PS-TKA) between December 2008 and April 2021 were identified in our joint replacement registration system. Two patients were excluded due to insufficient follow-up time (less than 2 years) and one was lost during follow-up. Thus, 30 patients (60 hips and 60 knees) were enrolled in the study, including 27 women (90%). The mean age of subjects at the time of their first arthroplasty was 47.2 years (range, 26–79), and their mean body mass index was 21 kg/m2 (range, 14.5–33.3). The mean disease duration at the first procedure was 16 years (range, 1–43), and the mean time between the first and last replacements was 27 months (Table 2). BTHKA was indicated only in patients who meet the following three criteria: (1) strong motivation for restoration of function, correction of deformities, or relief of pain; (2) the ASA grade ≤ 3; and (3) the activity of RA can be controlled by medications.

**Table 2:** Demographic baseline data

Variable	Demographics of patients
Age (years)^a^	47.2 (26–79)
Sex	
Male	3 (10%)
Female	27 (90%)
Height (cm)^a^	153.8 (135–168)
Weight (kg)	49.7 (34–75)
Body mass index (kg/m^2^)^a^	21 (14.5–33.3)
Disease duration (years)^a^	16 (1–43)
Operation interval (months) ^b^	27 (1–112)
Follow-up (months)	84 (24–156)

^a^data from the first arthroplasty

^b^the interval between the first and last arthroplasty

### Surgical procedures and perioperative regimens

All operations were performed under general anesthesia by five senior surgeons at our institution. THA was performed using the posterolateral approach with the patient in the lateral decubitus position, while TKA was performed using the medial parapatellar approach with the patient in the supine position. During THA, metal-on-metal, metal-on-polyethylene, ceramic-on-polyethylene, or ceramic-on-ceramic wear bearing materials were used. Cementless porous-coated acetabular and stem components (DePuy, Warsaw, IN, USA) were inserted using the press-fit technique. In TKA, a cemented posterior-stabilized fixed-bearing prothesis and a polyethylene insert (DePuy or Stryker, Mahwah, NJ, USA) were implanted. Fixed knee flexion deformities were corrected using bone resection and soft tissue release. Postoperatively, all received prophylactic broad-spectrum antibiotics and low-molecular-weight heparin antithrombotic therapy. And non-steroidal anti-inflammatory drugs (NSAIDs) were used to relieve pain and reduce the possibility of heterotopic ossification (HO). In the early postoperative period, patients performed isometric exercises and positive motion exercises in bed under the guidance of nurses and rehabilitation therapists. Continuous passive motion was required in some patients. After a comprehensive evaluation, patients were allowed to partial weight-bearing exercises with the help of the walker aid, then exercise with the help of cane and full weight-bearing exercises without help. During and after hospitalization, all patients continue to manage RA according to their original treatment regimens. Seven patients (23.3%) were treated with biological agents, six (20%) with glucocorticoids, and 21 (70%) with disease-modifying antirheumatic drugs.

### Outcomes measures

Routine examinations were performed preoperatively as well as at 3 and 6 months after surgery and then annually until the final follow-up. Clinical evaluations were conducted involving range of motion (ROM), and overall functional outcomes. Hip and knee ROM was measured with the patient in the supine position using a special ruler; flexion, flexion contracture, and abduction of the hip were measured, as well as flexion and flexion contracture of the knee. Overall functional outcomes, including the use of walking aids, walking distance, and ability to climb stairs, were assessed at the last follow-up. Patient-reported outcomes were measured using the Harris Hip Score (HHS) [13], Knee Society Score (KSS) [14], Western Ontario and McMaster Universities Index of Osteoarthritis (WOMAC) [15], and Patient Satisfaction Scale [16]. The HHS score, which includes four domains (pain, function, deformity, and ROM), was developed to assess the outcomes of hip surgery and is commonly used to evaluate various hip disabilities and treatment methods. The KSS, comprising clinical (pain, stability, and ROM) and functional scores, is used to assess the outcomes of TKA. The WOMAC hip and knee scores include three subscales: pain, stiffness, and physical function. The higher WOMAC scores indicate severe pain and stiffness and impaired physical function, whereas 0 score is associated with better hip and knee conditions. The items of the Patient Satisfaction Scale, for which the overall score can range from 0 to 100, include patients’ overall satisfaction with surgery, extent of pain relief, and ability to perform work and/or recreational activities. Higher scores are associated with greater self-reported satisfaction.

Full-length standing images and radiographs of all joints were obtained at the last follow-up by two researchers not involved in surgical procedures. The radiolucent lines of the seven zones around the femoral component and the three zones around the acetabular side were also defined based on the literature [17, 18]. Loosening and failure of TKA were analyzed using the Knee Society Roentgenographic Scoring System [19]. A radiographically loose component was defined as a radiolucent line > 2 mm around the entire circumference of the prosthesis, subsidence of the prosthesis, or a change in alignment from a previous radiograph [5]. Throughout the study, complications were diagnosed based on clinical examination and radiography.

### Statistical analysis

Statistical analysis was performed using SPSS 26.0 (IBM, Chicago, IL, USA). Continuous data was shown as means with ranges, while categorical data was shown as numbers and percentages. Differences between pre- and postoperative measurements were assessed for significance using a two-sided paired t-test, and those associated with *P* < 0.05 were considered statistically significant. Kaplan-Meier survival analysis [20] was used to estimate the relationship between implant failure-free survival and time after surgery. Implant failure was defined as loosening or performance of revision surgery for any reason.

## Results

The mean follow-up was 84 months (24-156) (Table 2). The sequence and interval of operation were presented in Table 3. Comparison of the pre- and postoperative ROM showed that motion was significantly better at the last postoperative follow-up than at baseline, and that the mean preoperative combined hip and knee flexion increased to 218.6° (125–260°). HHS pain, function and total scores at last follow-up increased significantly from 11.3 points (0-20), 6.2 points (0-16), and 23.2 points (4-44) points preoperatively to 43.8 points (40-44), 32.6 points (7-44), and 84.5 points (58-96), respectively (*P* < 0.001). KSS pain, stability, total clinical, and total function scores improved significantly from 11.4 points (0-20), 22.4 points (10-25), 33.6 points (8-57), and 5.4 points (0-25) points preoperatively to 48.7 points (40-50), 25 points (25-25), 94.5 points (81-100), and 74.5 points (0-100) at last review, respectively (*P* < 0.001). WOMAC hip and knee scores also improved significantly (*P*<0.001). At last follow-up, overall satisfaction scores were 92.5 after THA and 89.6 after TKA (Table 4).In addition, we found that the use of walking aids was limited to 11 patients (36.7%) after surgery, and none of the patients was confined to a bed or wheelchair. All patients improved in their walking distance, with six (20%) achieving an unlimited distance. Nearly all patients (96.7%) improved in their ability to climb stairs, and 17 (56.7%) were able to climb stairs without any aids at the last follow-up (Table 5).

**Table 3:** The sequence and interval of operation

Number	Sequence	Interval (months)	Number	Sequence	Interval (months)
1	RH + RK → LH + LK	1	16	LH → LK → RH → RK	1 → 7 → 5
2	RK → LK → LH → RH	1 → 30 → 9	17	LK → RK → RH → LH	1 → 84 → 2
3	LH + LK → RH + RK	1	18	RH → RK → LK → LH	1 → 1 → 108
4	RH → LH → LK → RK	1 → 1 → 2	19	LH → RH → RK → LK	1 → 36 → 76
5	LH → RH → RK → LK	1 → 2 → 2	20	RH → RK → LH → LK	2 → 2 → 43
6	RH → LH → RK → LK	1 → 1 → 2	21	RH → LH → RK → LK	1 → 3 → 21
7	RH → LH → LK → RK	1 → 38 → 3	22	LH → RH → LK → RK	2 → 29 → 3
8	LH → RK → LK → RH	12 → 1 → 36	23	RH → LH → LK → RK	1 → 1 → 10
9	LH → RH → RK → LK	1 → 11 → 4	24	LK → LH → RH → RK	2 → 9 → 4
10	LK → LH → RH → RK	5 → 8 → 2	25	RH → LH → LK → RK	9 → 6 → 4
11	LH → RH → LK → RK	2 → 11 → 36	26	LH → RH → LK → RK	2 → 13 → 36
12	LH → RH → LK → RK	1 → 2 → 1	27	LH → RH → RK → LK	2 → 2 → 1
13	LH + LK → RH + RK	3	28	LH + LK → RH + RK	5
14	RH → LH → LK → RK	4 → 7 → 2	29	LH → RH → LK → RK	3 → 8 → 2
15	LH → RH → LK → RK	1 → 2 → 2	30	LH → RH → LK → RK	4 → 4 → 6

*RH* Right hip, *RK* Right knee, *LH* Left hip, *LK* Left knee

**Table 4:** Preoperative and postoperative range of motion and patient-reported outcomes

Variable	Before surgery	Last follow-up	*P* value
Hip			
Flexion, °	66.5 (0–110)	114.5 (80–135)	<0.001
Flexion contracture, °	7.8 (0–60)	0 (0–0)	<0.001
Abduction, °	10.4 (0–40)	40.2 (25–50)	<0.001
WOMAC score			
Pain	10.8 (8–13)	0.2 (0–3)	<0.001
Function	50.2 (30–68)	16.8 (6–52)	<0.001
Total	63.3 (39–85)	16.9 (6–52)	<0.001
HHS score			
Pain	11.3 (0–20)	43.8 (40–44)	<0.001
Function	6.2 (0–16)	32.6 (7–44)	<0.001
Total	23.2 (4–44)	84.5 (58–96)	<0.001
Satisfaction score			
Overall		92.5 (50–100)	
Pain		98.8 (75–100)	
Function		86.3 (25–100)	
Recreation		84.6 (25–100)	
Knee			
Flexion, °	83.7 (0–130)	104.1 (35–130)	<0.001
Flexion contracture, °	19.6 (0–77)	0.5 (0–10)	<0.001
WOMAC score			
Pain	10.6 (8–14)	0.3 (0–3)	<0.001
Function	47.7 (32–68)	17.8 (7–53)	<0.001
Total	60.3 (42–86)	18.2 (7–55)	<0.001
KSS clinical score			
Pain	11.4 (0–20)	48.7 (40–50)	<0.001
Total	33.6 (8–57)	94.5 (81–100)	<0.001
KSS function score			
Total	5.4 (0–25)	74.5 (0–100)	<0.001
Satisfaction score			
Overall		89.6 (50–100)	
Pain		96.3 (75–100)	
Function		85 (25–100)	
Recreation		82.5 (25–100)	
Combined flexion, °	150.2 (0–220)	218.6 (125–260)	<0.001

*WOMAC* Western Ontario and McMaster Universities Index of Osteoarthritis, *HHS* Harris Hip Score, *KSS* Knee Society Score

**Table 5:** Preoperative and postoperative overall functional outcomes

Variable	Before surgery	Last follow-up
Walking aids, n (%)		
None	0 (0%)	19 (63.3%)
One cane/crutch	2 (6.7%)	8 (26.7%)
Two canes/crutches	9 (30%)	1 (3.3%)
Rollator walker	2 (6.7%)	2 (6.7%)
Bed/wheelchair	17 (56.7%)	0 (0%)
Walking distance, n (%)		
Unlimited	0 (0%)	6 (20%)
> 2 km	0 (0%)	8 (26.7%)
1–2 km	0 (0%)	10 (33.3%)
0.5–1 km	5 (16.7%)	4 (13.3%)
< 0.5 km	14 (46.7%)	2 (6.7%)
unable	11 (36.7)	0 (0%)
Stairs, n (%)		
Normal	0 (0%)	17 (56.7%)
Banister	1 (3.3%)	11 (36.7)
Any fashion	8 (26.7%)	1 (3.3%)
Unable	21 (70%)	1 (3.3%)

**Fig. 1:**
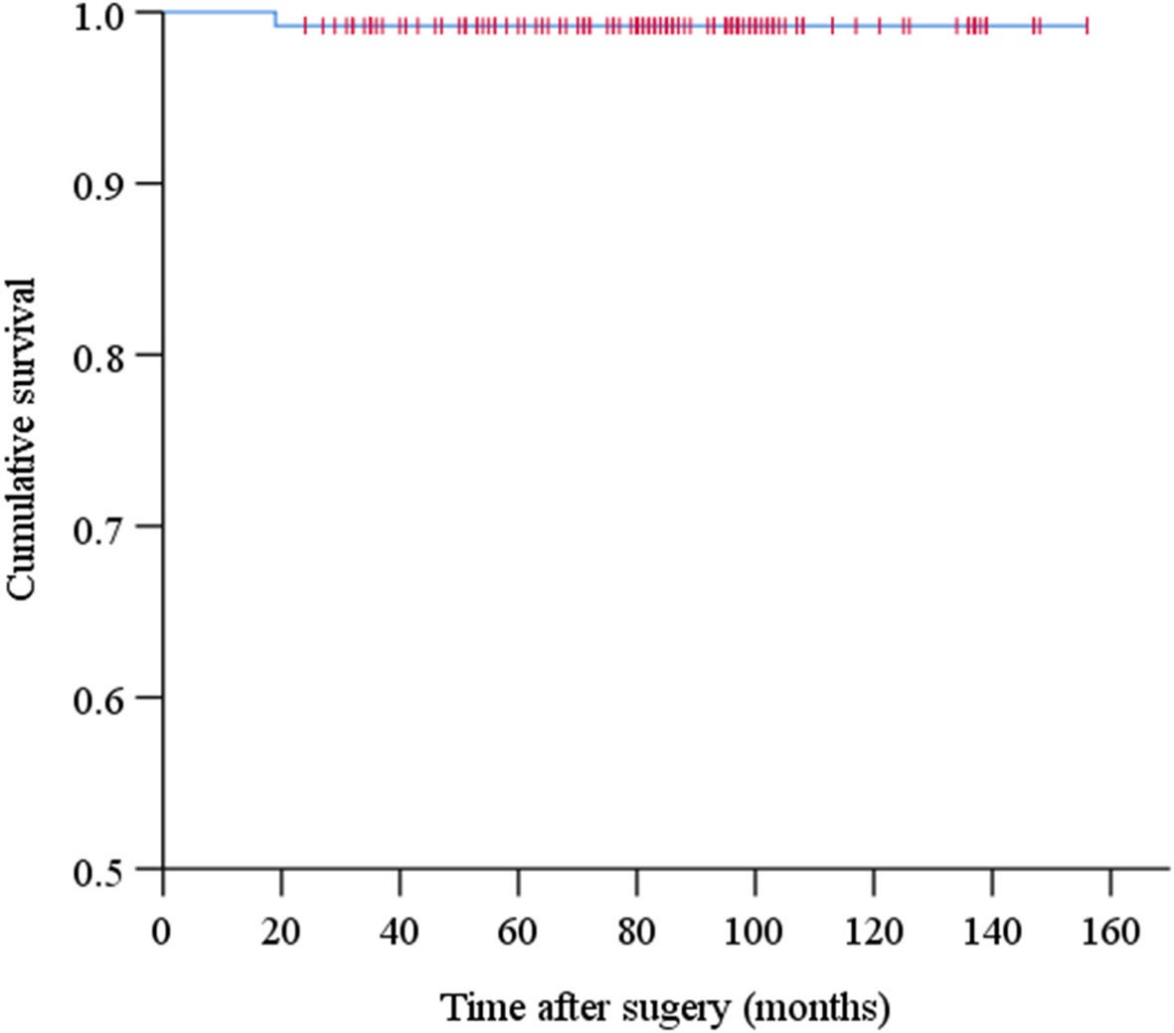
Kaplan-Meier survival curve of implant survival, indicating how long before implant loosening or any-cause revision surgery occurred

During the follow-up, one hip developed postoperative prosthesis dislocation. Four knees occurred complications: one superficial wound infection, two delayed wound healing, and one knee instability. All complications can be treated medically or surgically. Kaplan-Meier analysis indicated that 99.2% of implants (95% confidence interval, 94.4–99.9%) survived to the mean follow-up of 84 months without loosening or revision surgery (Fig. 1).

**Fig. 2:**
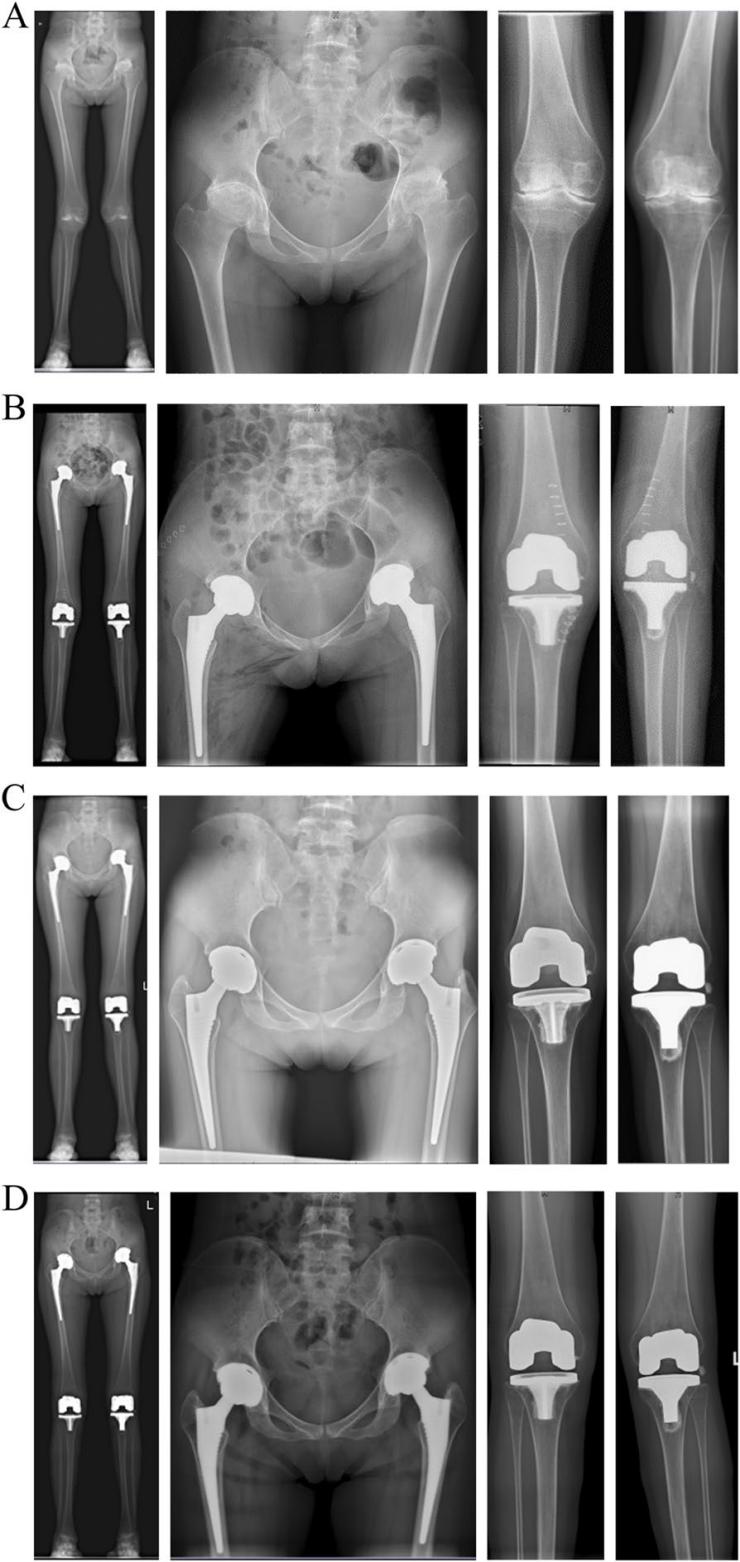
Radiographs of a 26-year-old woman with 16-year rheumatoid arthritis who underwent bilateral cementless total hip arthroplasty and cemented posterior-stabilized total knee arthroplasty. The operation sequence was left knee, left hip, right hip, and right knee, with the surgeries separated by five, eight, and two months. **A** Preoperative radiographs show uniform joint space narrowing in the bilateral hips and knees. **B** Early postoperative radiographs. **C**, **D** Postoperative radiographs were taken at the mean of 62 and 108 months, showing all components to be stable

At the last follow-up, asymptomatic HO was seen in two hips: there were one of Brooker grade I and one of Brooker grade II. A radiolucent line < 1 mm was detected around the acetabular cup in two hips in zone III and in one hip in zones I and III. Moreover, three knees in the medial tibial plateau and one knee in the lateral tibial plateau showed a radiolucent line < 1 mm around the tibial component. Figure 2 showed hips and knees were radiographically stable in a satisfactory position during the mean 9-year follow-up.

## Discussion

In this study, we examined the clinical, patient-reported, and radiographic outcomes of 30 RA patients who underwent bilateral cementless THA and cemented PS-TKA and who were followed up for a mean of 84 months. To our knowledge, our work is the largest cohort study reporting mid- to long-term results of relatively uniform BTHKA in RA patients, with a minimum follow-up of 2 years.

The sequence and interval of operation are uncertain in our study, which is consistent with other published studies [8]. We agree the opinion [7, 8] that the joint with the most severe symptom was replaced first, and when possible, ipsilateral hip arthroplasty preceded knee arthroplasty. It is important to note that extreme hip flexion should be avoided during knee replacement, as it may result in dislocation of the replaced hip [8], despite no hip dislocation occurred during surgery in this study. The sequence and interval of replacement depends mainly on the sequence and interval of joint involvement. However, we found that only a minority of patients, at first presentation, obviously require both hips and knees replaced. This can cause the different orders of arthroplasty. Studies have reported that one-stage ipsilateral hip and knee replacement is a good option for patients with severe deformities and contractures of ipsilateral joints [10, 21, 22]. These cases may be considered for one-stage ipsilateral hip and knee replacement if replacement of only one joint will not allow the patient to achieve a straight leg and early weight bearing. However, there is concern about the morbidity and mortality in these cases due to the extent of the surgery. The extensive surgery may result in excessive swelling, and increased rates of deep vein thrombosis and pulmonary embolus. In addition, the post-operative rehabilitation may be challenging given their contractures, systemic disease and muscle atrophy. There are four patients of our study whose left hip and knee were done in the same operation, in order to correct their contractures and allow early weight bearing after surgery. They did well and did not encounter any complications. Nevertheless, the surgeon must be aware of the increased morbidity and mortality of the procedures. We think that adequate preoperative evaluation, experienced surgeons and good perioperative management are important to ensure the safety of patients.

One of the most concerns of RA patients receiving BTHKA is postoperative function. Previous studies have shown that RA patients with combined hip and knee flexion > 190° can maximize functional outcomes [3, 23]. This is consistent with our routine clinical practice to help patients achieve greater postoperative ROM as much as possible. In the present study, postoperative ROM and overall functional outcomes were significantly better than in the preoperative values and in earlier reports [3, 6–11]. We speculate that the main possible reason, as Jergesen et al. expected [6], is advances in surgical technique and prosthesis design that make most patients toward the attainment of combined hip and knee flexion in excess of 190°, which contributed to the good overall functional outcomes. Twenty-eight patients (93.3%) showed combined hip and knee flexion > 190° at the last follow-up, while 27 (96.4%) were able to climb stairs independently and walk more than 500 m. Although two patients in our cohort walked less than 500 m, required rollator walkers most of the time, and could not climb stairs independently, both patients showed better overall function at last follow-up than preoperatively, and they reported high satisfaction with pain relief in the replaced joints.

PROMs provide a standardized method to assess important, subjective health status information that can’t be detected by objective or surgeon-reported outcome measures [24]. Multiple studies have shown that HHS, KSS, and WOMAC scores are reliable PROMs for hip and knee joint replacement [14, 25–27]. Actually, two preliminary studies have used HHS score to evaluate the effectiveness of BTHKA in RA patients showed that the mean score increased by ~ 54 points after surgery [8, 11]. However, HHS is a hip-specific score and is not a good measure of outcome for TKA. Here, in addition to using HHS score, KSS and WOMAC scores were also applied to evaluate preoperative and postoperative patient-reported outcomes. We found that all three scores were significantly better at last follow-up and that the mean postoperative HHS, KSS clinical, KSS functional scores were 61.3, 60.9, 69.1 points higher than the preoperative value. We believe that satisfactory patient-reported outcomes postoperatively in this study should also be closely related to excellent ROM [28, 29].

Residual pain after total joint replacement remains a concern: 8–20% of patients undergoing TKA or THA complain of unexplained residual pain [30, 31]. In fact, up to 50% of RA patients who undergo BTHKA report moderate or severe residual pain at final follow-up, especially in the replaced knee [6, 7, 9, 10]. In our study, postoperative pain improved significantly in all hips and knees based on the HHS, KSS and WOMAC pain scores. Although residual pain occurred in 5% of the hips and 11.6% of the knees, all pain were rated as mild.

Earlier studies have identified aseptic loosening as the most common cause of revision surgery. In the study of McDonald, with a mean follow-up of 25 months, four of fifty-two (7.7%) knees underwent revision for aseptic loosening [7]. Similarly, one of forty (2.5%) knees was revised due to loose tibia component after 8 years, and two of forty (5%) hips were reoperated due to aseptic loosening after and 12 years [6]. However, none of our patients developed aseptic loosening in our cohort. More importantly, all replaced hips and knees were radiographically stable by the assessment of the radiolucent line.

Obtaining postoperative stability is also key in joint replacement [32], which is particularly important in RA patients, which often involves medial and lateral collateral ligaments and other soft tissues. Among our patients, six patients (eight knees) required ligament reconstruction and three patients (three knees) required implantation of constrained condylar knee prostheses. However, only one required revision due to knee instability that developed progressively during 19 months surgery, probably because a thin insert was used and a varus developed in the ankle, leading to laxity and uneven stress in the knee joint. In this case, we replaced the original insert with a thicker one and simultaneously performed ankle fusion. At final review, the revision knee is stable based on KSS.

Overall, bilateral cementless THA and cemented PS-TKA provide reliable outcomes for RA patients within a mean follow-up time of 84 months, which can be mainly attributed to advances in prosthesis design and corresponding surgical techniques. Meanwhile, patients reported high levels of satisfaction with surgery, extent of pain relief, and ability to perform work and/or recreational activities.

The present study has some advantages over previous analyses of BTHKA because of our relatively large sample size, long follow-up, and the use of validated PROMs, and because the patients achieved reliable outcomes. Radiographic assessments have also proven to be useful in our study, because patients with complications may be asymptomatic [33]. Meanwhile, postoperative radiographs by measuring the periprosthetic radiolucent line are the more established method of assessing implant stability [34]. On the other hand, our study also had certain limitations, such as the fact that it was retrospective and no comparison group was involved. Moreover, knee implants are provided by two companies and the designs are slightly different. But they are both posterior-stabilized fixed- bearing prostheses and are currently still widely used in the TKA. There may have been heterogeneity in our results because five surgeons performed all operations; however, all used the standard surgical techniques, and surgical plans were agreed among all surgeons during routine preoperative meetings.

## Conclusions

Our study suggests that bilateral cementless THA and cemented PS-TKA provides reliable mid-long-term clinical, patient-reported, and radiographic outcomes in RA patients, with a low risk of revision and high patient satisfaction. Further follow-up of this cohort of RA patients is planned in order to analyze the long-term outcomes.

## Data Availability

Public access to the database is closed. For us, all related datasets were permitted to access and use by the Clinical Trials and Biomedical Ethics Committee of West China Hospital, Sichuan University. And the datasets used and/or analyzed during the current study are available from the corresponding author on reasonable request.

## Abbreviations

THA: Total hip arthroplasty
TKA: Total knee arthroplasty
BTHKA: Bilateral total hip arthroplasty and total knee arthroplasty
PROMs: Patient-reported outcomes measures
PS-TKA: Posterior-stabilized total knee arthroplasty
HO: Heterotopic ossification
ROM: Range of motion
HHS: Harris Hip Score
KSS: Knee Society Score
WOMAC: Western Ontario and McMaster Universities Index of Osteoarthritis
